# Merlin deficiency supports an immunosuppressive milieu in breast cancer

**DOI:** 10.1038/s41420-026-03223-7

**Published:** 2026-07-09

**Authors:** Mohamed H. Elbahoty, Brandon J. Metge, Amr R. Elhamamsy, Ian Miranda, Melanie Aldridge, Bohan Ning, Hua Guo, Dongquan Chen, Lalita A. Shevde

**Affiliations:** 1 Department of Pathology, University of Alabama at Birmingham, Birmingham, AL USA; 2 Medical Scientist Training Program, University of Alabama at Birmingham, Birmingham, AL USA; 3 Division of General Internal Medicine and Population Science, Department of Medicine, University of Alabama at Birmingham, Birmingham, AL USA; 4 O’Neal Comprehensive Cancer Center, University of Alabama at Birmingham, Birmingham, AL USA

**Keywords:** Cancer, Breast cancer

## Abstract

Despite substantial advances in early detection and therapeutic strategies, breast cancer remains a major cause of cancer-related morbidity and mortality worldwide. Changes in cytoskeletal proteins that typically regulate cellular architecture underpin tumor progression. Merlin, encoded by the *NF2* gene and a member of the ezrin–radixin–moesin protein family, functions as a critical cytoskeletal linker protein and a tumor suppressor that governs multiple oncogenic pathways and restricts epithelial–mesenchymal plasticity. While Merlin deficiency alters cellular architecture, the precise impact of a Merlin-deficient breast tumor on remodeling its immune microenvironment remains largely undefined. In this study, we demonstrate that in breast cancer, Merlin deficiency displays attributes consistent with enhanced epithelial–mesenchymal plasticity and enrichment of Hedgehog signaling. In vivo, Merlin-deficient tumors displayed accelerated growth and an immunosuppressive milieu characterized by increased abundance of regulatory T cells and M2-like macrophages. Treatment with Vismodegib, a pharmacological Hedgehog pathway inhibitor, reduced tumor growth of Merlin-deficient basal-like tumors, accompanied by a decrease in tumor-infiltrating regulatory T cells and immunosuppressive M2-like macrophages, and increased recruitment of pro-inflammatory M1-like macrophages. The data present Hedgehog inhibition as a targetable vulnerability of Merlin-deficient basal-like, triple-negative breast cancer.

## Introduction

Despite recent advances in early detection and treatment approaches, including immunotherapy [1], breast cancer remains a major concern due to its heterogeneity and the development of drug resistance. Understanding the molecular underpinnings that drive breast cancer initiation, progression, and metastasis is crucial for the development of more effective and targeted therapies [2].

Tumor growth and metastasis warrant dynamic cytoskeletal changes that enable cancer cells to alter morphology, detach from the primary tumor, invade tissues, and infiltrate distant organs. These changes underpin essential activities such as cell motility, invasion, intravasation, and extravasation [3]. ERM (Ezrin, Radixin, and Moesin) proteins are cytoskeletal linker proteins that connect the plasma membrane to the actin cytoskeleton, regulating cell shape, adhesion, and motility. ERM proteins have numerous crucial functions in tumor development and metastasis, including promoting cytoskeletal remodeling, altering cell polarity, facilitating migratory and invasive behaviors, and activating oncogenic signaling pathways [4]. Merlin, also known as neurofibromin 2 (NF2), is a critical member of the ERM protein family that functions as an important tumor suppressor. First discovered for its involvement in neurofibromatosis type II [5], Merlin modulates a variety of cellular processes, including proliferation, adhesion, and migration, by modifying critical signaling, such as Hippo, PI3K/AKT, receptor tyrosine kinase, and Hedgehog signaling pathways [6, 7].

Merlin interacts with the actin cytoskeleton and membrane-associated proteins to control cellular architecture and polarity; its loss disrupts these structural programs, thus licensing stemness, epithelial–mesenchymal transition (EMT), invasion, and dissemination [8, 9]. In breast cancer, Merlin deficiency is associated with enhanced tumor growth, increased metastatic capacity, and treatment resistance [10], and Merlin loss impairs Hippo pathway control, thereby enhancing oncogenic gene expression [7]. Loss of Merlin has been linked to metastasis, with reduced Merlin expression observed in metastatic breast lesions compared to primary tumors [11]. Recent research suggests that Merlin loss may also influence the tumor microenvironment (TME) [12–14] and increase breast cancer heterogeneity [15]. As such, understanding how Merlin deficiency promotes tumor progression may reveal actionable vulnerabilities for precision therapy. However, no targeted treatments currently exist for malignancies driven by Merlin loss, particularly in breast cancer [16, 17]. In this study, we demonstrate that Merlin loss exacerbates breast cancer aggressiveness by licensing EMT-driven progression, enabling immune evasion, and shaping an immune-suppressive microenvironment, while revealing inhibition of Hedgehog signaling as a promising therapeutic strategy for Merlin-deficient breast cancer.

## Results

### *NF2* loss in breast cancer is clinically frequent and predicts poor prognosis

We first explored the landscape and prognostic relevance of NF2 alterations across multiple cancer types using pan-cancer datasets. Across TCGA, *NF2* is altered at variable but generally low frequencies, with the highest rates observed in mesothelioma (MESO), uterine carcinosarcoma (UCS), and glioblastoma (GBM). These alterations are dominated by mutations and deep deletions, whereas amplifications are uncommon, consistent with Merlin’s role as a tumor suppressor. In breast cancer and several other malignancies, genomic alterations of *NF2* are relatively rare. Instead, *NF2* dysregulation appears to arise predominantly from reduced expression rather than coding mutations or structural variants (Supplementary Fig. 1A). Pan-cancer RPPA (Reverse Phase Protein Array) analyses mirrored these findings, revealing widespread NF2 downregulation across epithelial malignancies, including breast, prostate, and uterine cancers (Supplementary Fig. 1B). Pan-cancer survival analyses further demonstrated that NF2-deficient patients experienced significantly worse progression-free survival (HR = 0.804; *p* < 0.0001; Supplementary Fig. 1C) and overall survival (HR = 0.768; *p* < 0.0001; Supplementary Fig. 1D). Consistent with this, pan-cancer analysis of whole genomes (PCAWG) also revealed poorer overall survival in NF2-deficient patients (HR = 0.804; *p* < 0.0001; Supplementary Fig. 1E), reinforcing Merlin’s tumor-suppressive function.

**Fig. 1:**
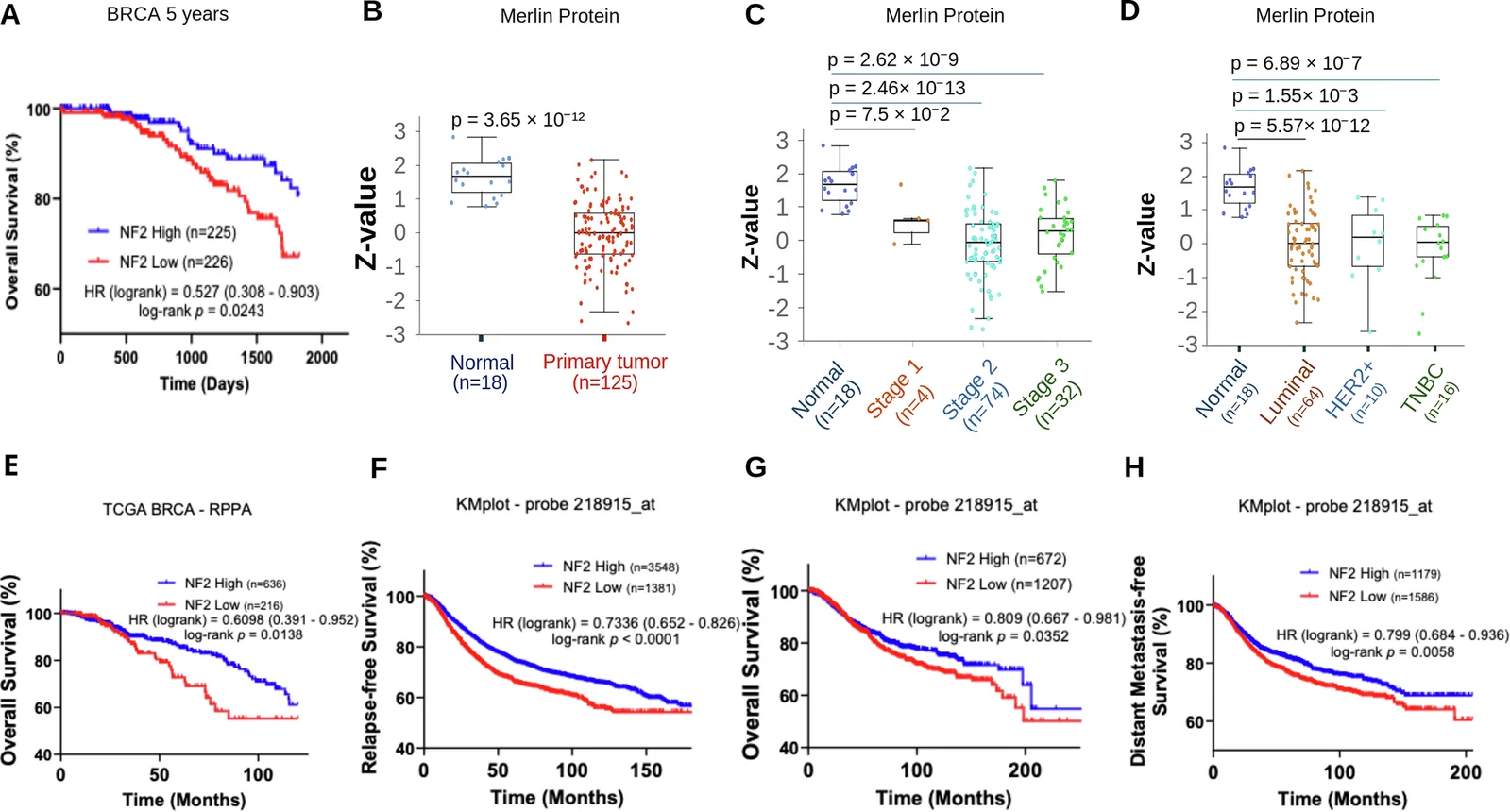
Merlin loss in breast tumors predicts poor prognosis, reduced expression, and adverse clinical outcomes. **A** Kaplan–Meier 5-year overall survival analysis of breast cancer patients from the TCGA RPPA dataset stratified by Merlin protein expression. Low Merlin expression (red, *n* = 226) was associated with significantly reduced survival compared with high Merlin expression (blue, *n* = 225) (hazard ratio [HR] = 0.527; 95% CI: 0.308–0.903; log-rank *P* = 0.0243). **B** Merlin protein levels were significantly lower in primary breast tumors (*n* = 125) than in normal breast tissues (*n* = 18) in the CPTAC dataset (*P* = 3.65 × 10⁻¹²). **C** Stage-specific analysis showed progressive Merlin downregulation from Stage 1 (*n* = 4) to Stage 3 (*n* = 32), with the greatest suppression observed at Stage 2 (*n* = 74) compared with normal tissues (*n* = 18) (*P* = 2.46 × 10⁻¹³). **D** Merlin expression was significantly reduced across all major molecular subtypes, luminal, HER2-positive, and triple-negative breast cancer (TNBC), relative to normal breast tissue. **E** Kaplan–Meier survival analysis of breast cancer patients from the TCGA RPPA dataset based on Merlin protein expression. Patients with low Merlin expression (red curve) exhibited significantly reduced overall survival compared to those with high expression (blue curve) (hazard ratio [HR] = 0.6098; 95% confidence interval [CI]: 0.391–0.952; log-rank *P* = 0.0138). The number of patients at risk is indicated below the x-axis. **F**–**H** Kaplan–Meier survival analyses evaluating the prognostic value of NF2 mRNA expression (probe 218915_at) across several survival endpoints in breast cancer patients. **F** Relapse-Free Survival (RFS): Patients with low NF2 mRNA expression (red curve) exhibited significantly shorter RFS compared to high expressers (blue), with a hazard ratio (HR) of 0.7336 (95% CI: 0.652–0.826; *P* < 0.0001), suggesting Merlin deficiency increases the risk of recurrence. **G** Overall Survival (OS): Low NF2 mRNA levels were associated with worse OS (HR = 0.809; 95% CI: 0.667–0.981; *P* = 0.0352). **H** Distant Metastasis-Free Survival (DMFS): Patients with low NF2 mRNA expression showed a higher likelihood of distant metastatic events (HR = 0.779; 95% CI: 0.684–0.936; *P* = 0.0058). In all panels, the blue curve represents patients with High Merlin/NF2 expression, and the red curve denotes low expression.

Next, we assessed the prognostic impact of NF2 in The Cancer Genome Atlas Breast Invasive Carcinoma (TCGA-BRCA) dataset. Low NF2 protein expression defined a poor-prognosis subset with significantly worse overall survival (OS; HR = 0.527; *p* = 0.0243; Fig. 1A). We then interrogated large-scale clinical datasets to characterize NF2 expression patterns in breast cancer. Analysis of CPTAC (Clinical Proteomic Tumor Analysis Consortium) data revealed that NF2 protein levels were significantly lower in primary breast tumors (*n* = 125) compared with normal breast tissue (*n* = 18; *p* = 3.65 × 10⁻¹²; Fig. 1B). Stage-specific analysis revealed progressive downregulation of NF2 from stage I through stage III, with the most pronounced loss observed in stages II and III (*p* = 2.46 × 10⁻¹³ and *p* = 2.62 × 10⁻⁹, respectively, Fig. 1C). Across all molecular subtypes, NF2 expression was significantly reduced (Fig. 1D) relative to normal breast.

Kaplan–Meier survival analysis of the TCGA-BRCA RPPA (Reverse Phase Protein Array) dataset further demonstrated that patients with low NF2 protein expression had significantly reduced overall survival compared to those with high NF2 expression (HR = 0.6098; log-rank *p* = 0.0138; Fig. 1E). Consistent with these observations, low NF2 expression also predicted poorer outcomes across multiple clinical endpoints in breast cancer cohorts: shorter relapse-free survival (HR = 0.7336; *p* < 0.0001; Fig. 1F), overall survival (HR = 0.809; *p* = 0.0352; Fig. 1G), distant metastasis-free survival (HR = 0.799; *p* = 0.0058**;** Fig. 1H), regional recurrence-free survival (HR = 0.696; *p* = 0.0023; Supplementary Fig. 2A), and inferior post-progression survival (HR = 0.355; *p* = 0.0396; Supplementary Fig. 2B).

To investigate the biological consequences of Merlin loss, we performed gene set enrichment analysis (GSEA) using transcriptomic data from breast tumors in the context of NF2 expression. Hallmark pathway analysis identified upregulation of EMT alongside key oncogenic programs, including MYC targets, mitotic spindle, E2F targets, glycolysis, and TNFα signaling via NF-κB [18, 19] (Supplementary Fig. 2C). Reactome GSEA demonstrated enrichment of pathways associated with MET-driven cell motility (Supplementary Fig. 2D), whereas KEGG GSEA revealed dysregulation of adherens junction organization and DNA homologous recombination (Supplementary Fig. 2E). Consistent with upregulated EMT signature in Hallmark, GSEA of the TCGA-BRCA dataset revealed significant enrichment of EMT-associated genes in low NF2 tumors (NES = 1.424, *p* = 0.0122) (Supplementary Fig. 2F). Collectively, these results suggest that loss of NF2 promotes transcriptional reprogramming that enhances invasive potential and oncogenic signaling in breast cancer.

### Merlin silencing induces transcriptomic reprogramming of oncogenic and immune-modulatory pathways

**Fig. 2:**
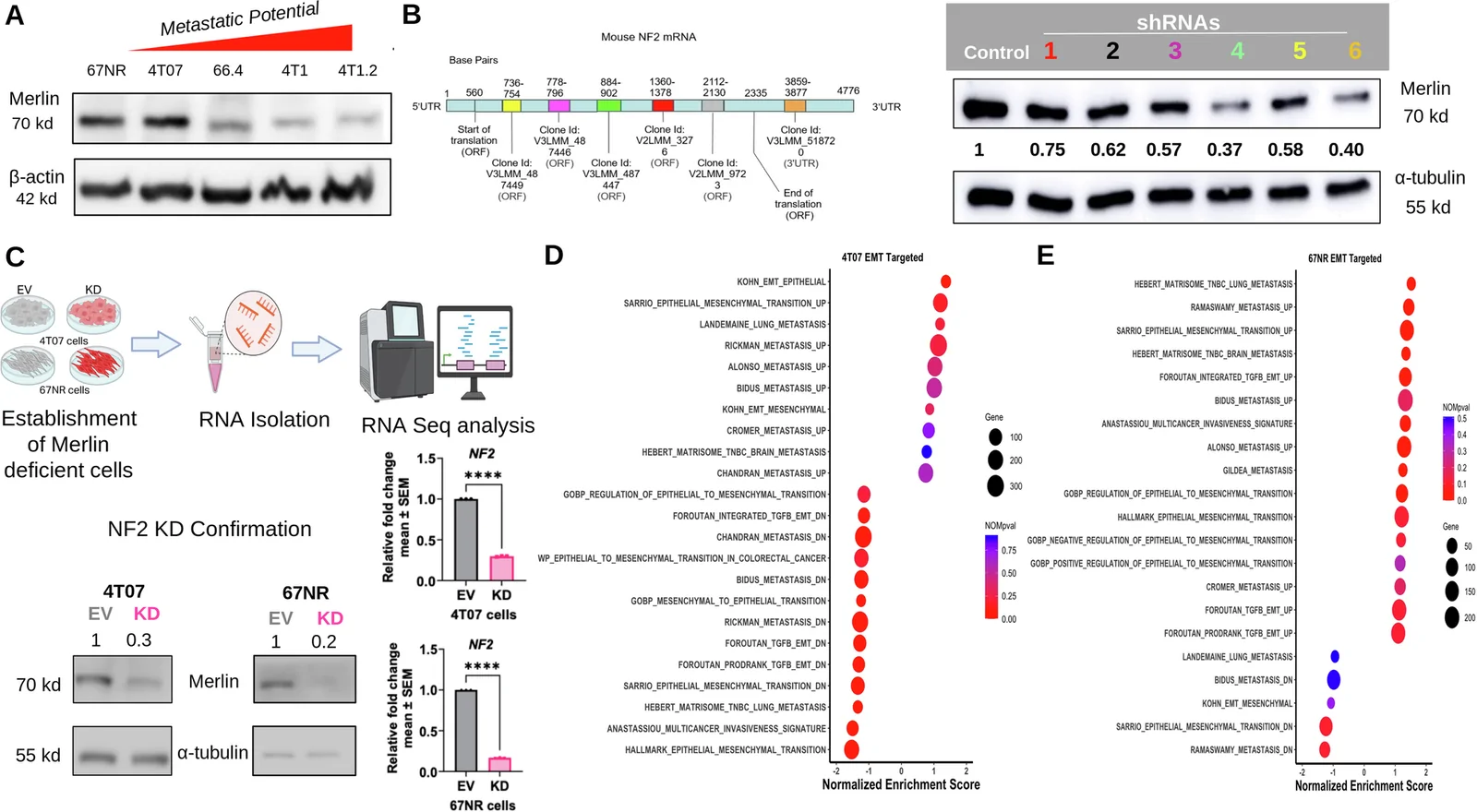
Establishment of Merlin-deficient breast cancer models and transcriptomic consequences of Merlin loss. **A** Representative immunoblots of baseline Merlin protein across a BALB/c mammary series (NMuMG → 67NR→4T07→66.4→4T1→4T1.2) arranged by increasing metastatic potential. **B** Schematic of mouse Nf2 mRNA indicating target sites for six pGIPZ shRNAs (colored boxes; ORF or 3′UTR). Screening in 4T07 cells of the six shRNAs (shRNA1–6) versus non-transfected control (C); Merlin and α-tubulin blots are shown with Merlin/tubulin ratios normalized to control. The clone shRNA4 (V3LMM_487447) produced the strongest and most reproducible knockdown and was advanced for model generation. **C** Schematic overview of experimental design. RNA isolation and bulk RNA sequencing were performed on Nf2 knockdown (KD) and empty vector (EV) control 4T07 and 67NR murine breast cancer cells to evaluate transcriptomic changes following Merlin loss. Nf2 silencing was validated by qRT-PCR and immunoblotting, confirming robust depletion of Merlin expression in both models (bar graphs, ****p < 0.0001). **D** Targeted EMT GSEA of RNA-seq data in 4T07 NF2-KD cells shows significant enrichment of epithelial–mesenchymal transition and multiple metastasis-associated gene signatures. **E** Targeted EMT GSEA of RNA-seq data in 67NR NF2-KD cells similarly demonstrates strong enrichment of EMT and pro-metastatic pathways, indicating a consistent induction of invasive transcriptional programs following Merlin loss. All quantitative data are presented as mean ± SEM. Statistical significance was determined using a two-tailed Student’s t-test for the 2^−ΔΔCt^ method for qRT-PCR analysis. Significance thresholds: ****p < 0.0001. The schematic overview in (**C**) was created using BioRender.com (BioRender Inc., 2025).

Given Merlin’s established roles in cytoskeletal regulation and tumor progression, we first assessed Merlin across a series of breast tumor cells spanning metastatic potential: non-metastatic (67NR), weakly metastatic (4T07), moderately metastatic (66cl.4), aggressive triple-negative (4T1), and their potently metastatic derivative (4T1.2) [20]. A stepwise decrement in Merlin expression was observed in association with increasing metastatic behavior: 66cl.4, 4T1, and 4T1.2, whereas Merlin protein levels in 67NR and 4T07 cells were comparable (Fig. 2A). Guided by these data, we proceeded with 4T07 and 67NR cells for targeted knockdown of Merlin. To engineer Merlin-deficient derivatives, we screened six pGIPZ mouse *Nf2* shRNAs spanning the ORF/3′UTR and identified clone V3LMM_487447 as the most potent and reproducible, achieving ~60–65% protein reduction versus non-transfected controls (Fig. 2B). This construct was advanced for stable line generation. We established matched *Nf2* knockdown and empty vector controls in 4T07 and 67NR cells (Fig. 2C). These pairs formed the foundation for all downstream functional, transcriptomic, and in vivo studies.

Building on pan-cancer and breast clinical analyses (Supplementary Figs. 1 and 2; Fig. 1), which linked low NF2 to progressive disease, poor survival, and enrichment of oncogenic and stemness programs, we next functionally characterized the impact of Merlin loss in our cell models. Bulk RNA-seq was performed in 4T07 and 67NR cells engineered to stably knockdown *Nf2* (KD) or empty vector (EV) (Fig. 2C). The knockdown was confirmed by qRT-PCR and immunoblotting (Fig. 2C).

Guided by the EMT signatures observed in our TCGA-BRCA analyses (Supplementary Fig. 2C, F), we interrogated our bulk RNA-seq datasets from 4T07 and 67NR *Nf2* KD cells for EMT-specific transcriptional changes. Targeted EMT GSEA demonstrated that Merlin loss in both 4T07 and 67NR cells led to significant enrichment of EMT signatures together with multiple metastasis-associated gene programs (Fig. 2D, E). Collectively, these results show that NF2 loss drives EMT-dominant transcriptional reprogramming, reinforcing Merlin’s essential role in restraining invasive progression.

Unsupervised clustering robustly separated KD from EV in both cell lines, underscoring broad transcriptomic reprogramming (Supplementary Fig. 3A, B). Canonical EMT markers were dysregulated: both 4T07 and 67NR KD cells presented with increased *Vim* (Vimentin) accompanied by changes in *Cdh1* (E-cadherin) and *Cdh2* (N-cadherin) (Supplementary Fig. 3C). Volcano plots (Supplementary Fig. 4A, B) followed by Hallmark GSEA revealed enrichment of MYC and E2F targets, cholesterol homeostasis, oxidative phosphorylation, KRAS signaling, and negative enrichment of pro-inflammatory networks (Supplementary Fig. 4C, D) [21–35]. These findings were reinforced with Reactome and KEGG analyses, revealing enrichment for cilium assembly, cholesterol biosynthesis, immune-modulatory pathways, and ECM organization/remodeling (Supplementary Fig. 5A–D).

### Merlin deficiency elicits an EMT program in breast cancer cells

**Fig. 3:**
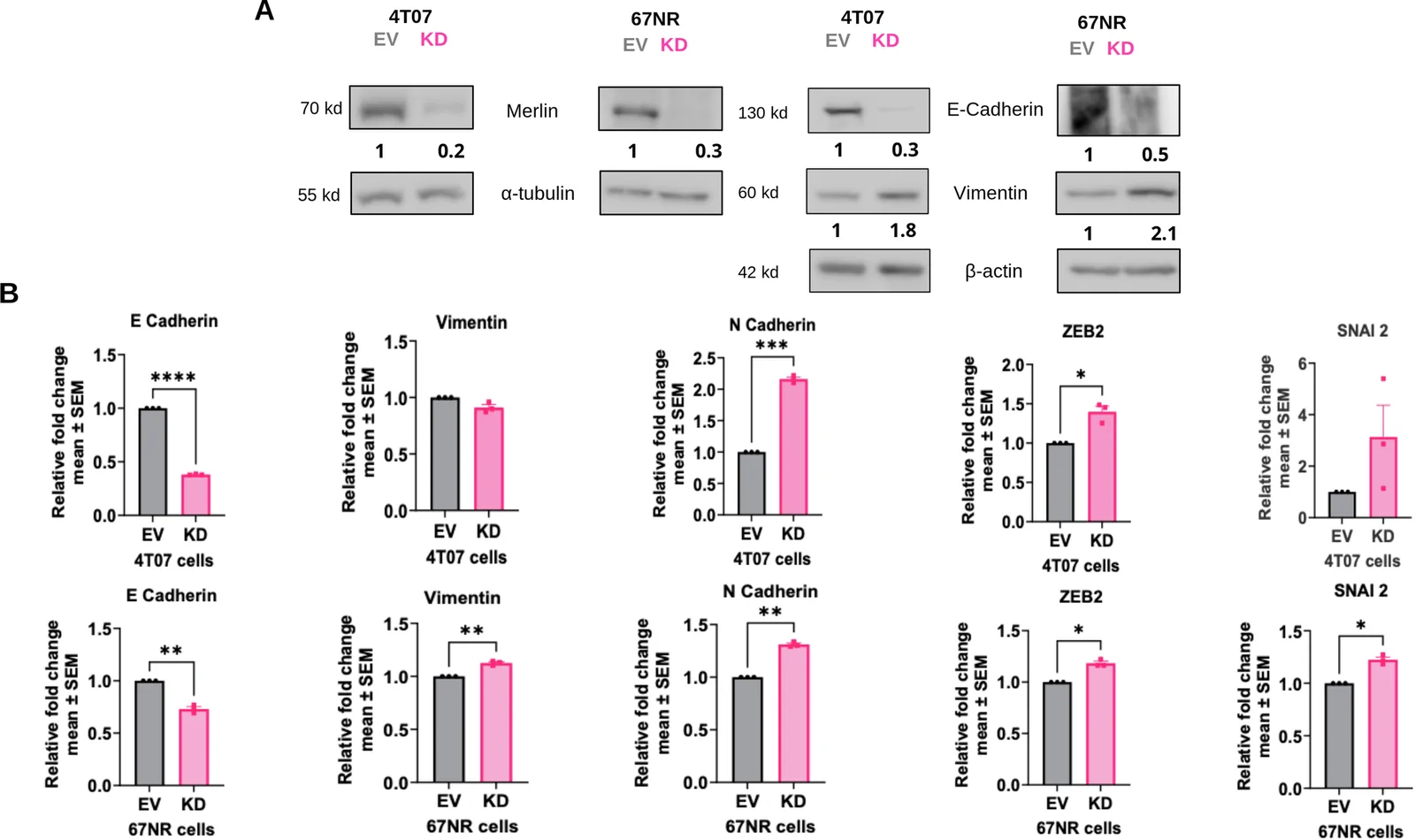
Epithelial–mesenchymal transition (EMT) induction following Merlin loss in 4T07 and 67NR breast cancer cells. **A** Representative immunoblot analysis showing efficient depletion of Merlin protein in Nf2 knockdown (KD) cells relative to empty vector (EV) controls in both models, with expression of Vimentin and E-cadherin. β-tubulin and β-actin were used as loading controls. **B** Quantitative RT-PCR (qRT-PCR) analysis further validated EMT markers dysregulation. All quantitative data are presented as mean ± SEM. Statistical significance was determined using a two-tailed Student’s t-test for the 2^−ΔΔCt^ method for qRT-PCR analysis. Significance thresholds: *p < 0.05, **p < 0.01, ***p < 0.001, ****p < 0.0001.

The transcriptional enrichment of EMT and ECM remodeling pathways observed in NF2-deficient human breast tumors (Supplementary Fig. 2) and our bulk RNA seq analysis (Supplementary Figs. 3–5) provided a compelling rationale to investigate EMT in our model systems. Merlin deficiency resulted in reduced E-cadherin and increased vimentin (VIM) protein expression in both model systems (Fig. 3A). To complement transcriptomic and immunoblotting analyses, we quantified the steady-state levels of EMT-associated transcripts. Both KD models registered decreased E-cadherin and upregulation of N-cadherin, ZEB2, and SNAI2. 67NR KD cells exhibited an increase in VIM (Fig. 3B), supporting a model-specific EMT response. An independent *NF2*-targeting shRNA validated similar impacts on EMT (Supplementary Fig. 6), cumulatively, informing that Merlin loss elicits an EMT program in breast cancer cells.

**Fig. 4:**
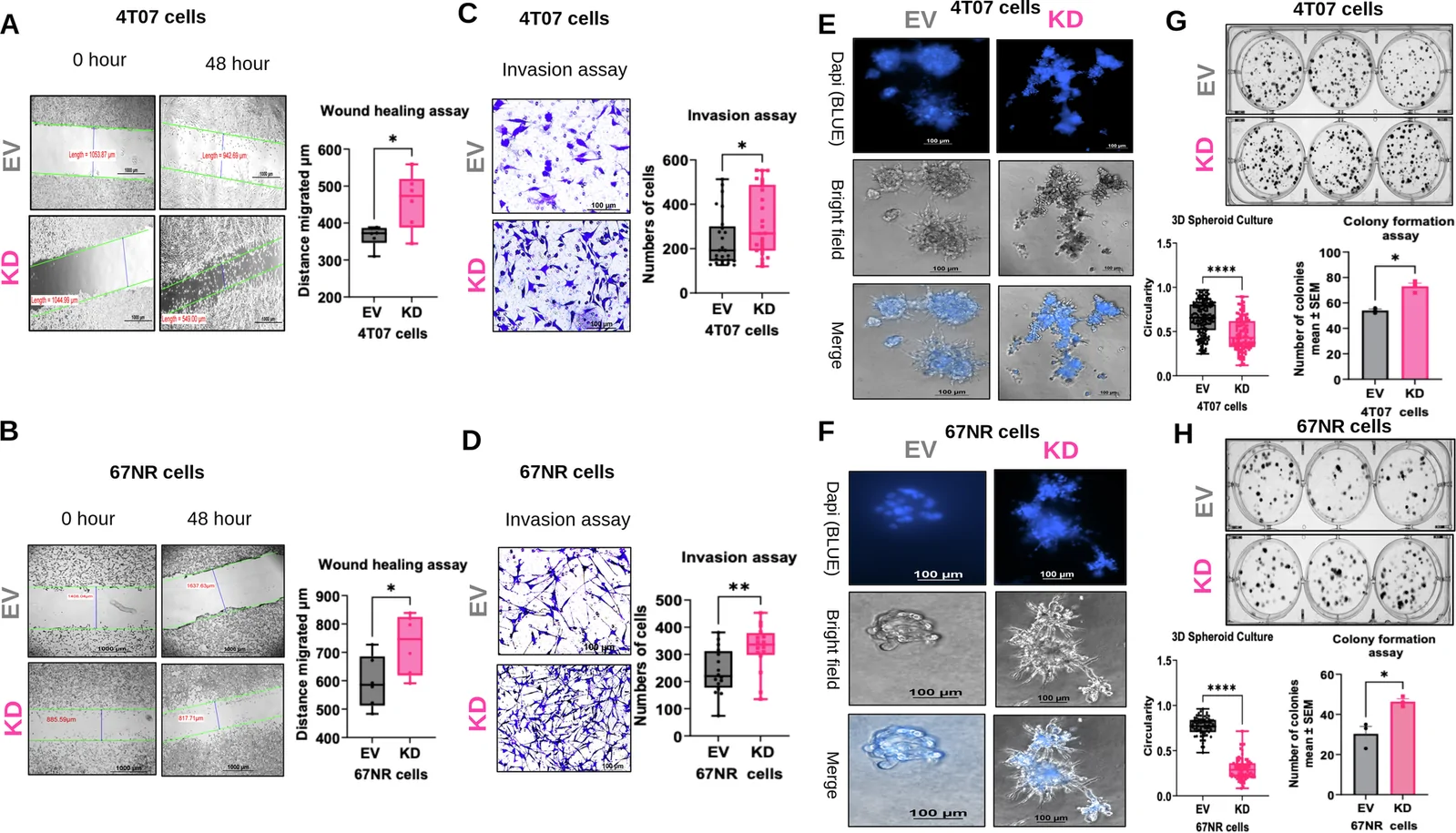
Merlin loss enhances migratory, invasive, and proliferative capacities in 4T07 and 67NR breast cancer cells. Wound healing assays: Representative phase-contrast images (6 fields) of 4T07 (**A**) and 67NR (**B**) cells at 0 and 48-h post-scratch show that Merlin knockdown (KD) cells exhibit significantly greater wound closure compared to empty vector (EV) controls, reflecting enhanced migratory potential. Quantification of the distance migrated is shown on the right. Invasion assays: Trans-well Matrigel invasion assays demonstrate a marked increase in invasive capacity in KD cells relative to EV cells for both 4T07 (**C**) and 67NR (**D**) lines. Representative crystal violet-stained membranes and quantification of invaded cells are shown (25 fields per group for 4T07 cells and 16 fields per group for 67NR cells). **E**, **F** 3D spheroid invasion: In 3D culture, KD spheroids exhibited more invasive morphologies characterized by reduced circularity and prominent radial protrusions. Images show DAPI nuclear staining (blue) and bright-field overlays for both 4T07 (**E**) and 67NR (**F**) spheroids. Quantitative analysis of colony circularity is shown (circularity was assessed in more than 100 colonies per group for 4T07 cells and more than 70 colonies per group for 67NR cells). Colony formation assays: KD cells formed significantly more colonies than EV cells after 10 days in adherent culture in both 4T07 (**G**) and 67NR (**H**) lines, indicating an enhanced proliferative phenotype. Representative plates and quantification of colony numbers are shown. Data are presented as mean ± SEM. Statistical comparisons were performed using unpaired two-tailed t-tests. *p < 0.05; **p < 0.01; ****p < 0.0001.

To characterize EMT-associated cellular behavior, we functionally queried the 4T07 and 67NR KD cells for migration and invasion. Merlin depletion accelerated wound closure in scratch assays, with KD cells out-migrating EV controls at 48 h in both models (Fig. 4A, B). In transwell assays, *Nf2* loss increased the invasive capacity of tumor cells (Fig. 4C, D), and in 3D growth conditions, the KD spheroids exhibited reduced circularity and prominent radial projections (Fig. 4E, F). In agreement with these attributes, supportive of enhanced oncogenic potential, KD cells formed significantly more colonies than EV (Fig. 4G, H). These phenotypic and functional changes were recapitulated with an independent shRNA targeting *Nf2* (Supplementary Fig. 7). The EV and Nf2-KD cells proliferated at comparable rates (data not shown), indicating that enhanced migration and invasion are reflective of an acquired program, independent of cell growth. Collectively, these assays demonstrate that Merlin loss elicited alterations functionally consistent with transcriptomic rewiring (Supplementary Figs. 3 and 4) and EMT induction (Fig. 3).

### Merlin deficiency enhances in vivo tumor growth and remodeling

**Fig. 5:**
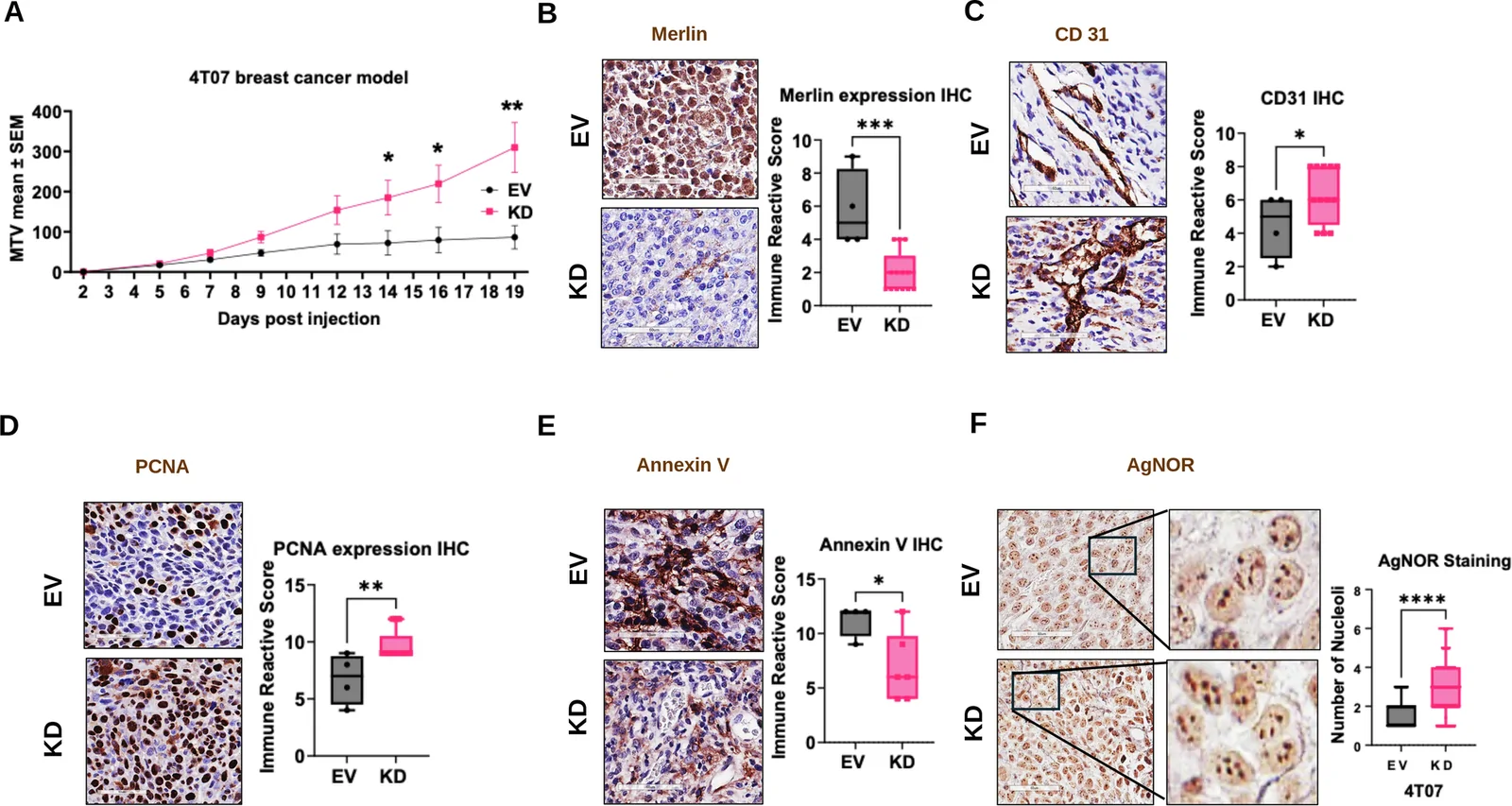
Immunohistochemical and in vivo studies show that Merlin loss promotes tumor growth, angiogenesis, proliferation, and apoptosis resistance in orthotopic 4T07 tumors. **A** Tumor growth kinetics of mice injected with control (EV, black) or Nf2 knockdown (KD, pink) 4T07 cells. Cells (70,000 per mouse) were orthotopically injected into the left inguinal mammary fat pad of female BALB/c mice (6–8 weeks old). Mean tumor volume (MTV) was measured at the indicated time points post-injection. **B** Immunohistochemical staining for Merlin demonstrates a marked reduction in brown chromogenic signal in Merlin knockdown (KD) tumors compared to empty vector (EV) controls, indicating effective in vivo silencing of Nf2. Quantitative analysis using the Immune Reactive Score (IRS) revealed a significant decrease in Merlin protein expression in KD tumors (***p < 0.001). **C** Staining for CD31, an endothelial marker, revealed enhanced vascularization in KD tumors, as shown by intensified chromogenic labeling of endothelial structures. IRS-based quantification confirmed a significant upregulation of CD31 expression in the absence of Merlin (*p* < 0.01), consistent with increased angiogenic activity. **D** IHC staining for PCNA, a proliferation marker, revealed stronger nuclear staining in KD tumors, indicative of increased mitotic activity. IRS quantification confirmed a significant elevation in PCNA expression in Merlin-deficient tumors (*p* < 0.01), suggesting accelerated tumor cell proliferation. **E** Annexin V immunolabeling demonstrated a reduction in apoptotic activity in KD tumors relative to controls. The observed decrease in Annexin V positive staining supports the role of Nf2 loss in conferring resistance to apoptosis, with IRS analysis showing a downward trend in KD tumors. **F** AgNOR staining (silver-stained nucleolar organizer regions) revealed increased nucleolar number in KD tumors, evidenced by a higher frequency of black-stained nucleoli per nucleus. Data are presented as mean ± SEM. Statistical comparisons were performed using appropriate tests; *p < 0.05; **p < 0.01; ***p < 0.001; ****p < 0.0001.

Building upon the evidence thus far and to model clinical manifestation in immunocompetent models, we established orthotopic mammary tumors using luciferase-labeled 4T07 or 67NR cells stably silenced for Merlin (KD) or transduced as empty vector controls (EV). Merlin depletion significantly accelerated tumor growth in both model systems, with KD tumors displaying greater mean tumor volumes (MTV) than EV controls (Fig. 5A, Supplementary Fig. 8A).

Tumor tissue analysis presented that Merlin loss (Fig. 5B, Supplementary Fig. 8B) was concordant with increased CD31⁺ microvessel density (Fig. 5C, Supplementary Fig. 8C) and higher PCNA positivity (Fig. 5D, Supplementary Fig. 8D), indicating enhanced angiogenesis and proliferation, respectively. In contrast, Annexin V staining was consistently reduced in KD tumors, supporting decreased apoptosis (Fig. 5E, Supplementary Fig. 8E). In order to pathologically ascribe such remarkable changes to enhanced aggressiveness and malignant potential, we assessed the tumor by Argyrophilic Nucleolar Organizer Region (AgNOR) staining. This stain identifies nucleoli that form around ribosomal DNA that is undergoing rapid transcription and is a pathological indicator of increased proliferation, malignancy, and metabolic activity. AgNOR counts were elevated in 4T07 KD tumors (Fig. 5F), indicative of higher tumor grade and proliferative activity [36–38]. 67NR KD tumors trended towards increased AgNOR staining, albeit not significant (Supplementary Fig. 8F); however, the nucleoli were highly irregular, indicative of heightened metabolic activity. These findings underscore a potential role for NF2/Merlin loss in permitting vascularization and malignant potential in vivo.

### Merlin deficiency perturbs Hedgehog signaling and imparts selective therapeutic vulnerability to Hedgehog pathway inhibition

Bulk RNA-seq analysis revealed that Merlin loss was associated with significant upregulation of cilium assembly programs and cholesterol biosynthesis (Reactome, Supplementary Fig. 5A). Given that the primary cilium serves as the central hub for Hedgehog (Hh) signaling, providing the platform for PTCH, SMO, and GLI regulation, enhanced ciliogenesis suggested the potential of activated Hh signaling. Prior work from our group demonstrated that Merlin loss-associated oxidative stress spurred Hh activation [7]. We performed GSEA of bulk RNA-seq data from 4T07 and 67NR models using multiple Hh-related gene sets curated in MSigDB. Both cell lines demonstrated enrichment of Hh signaling following *Nf2* knockdown, indicating pathway activation (Supplementary Fig. 9A, B). Aligned with these findings, 4T07 KD cells expressed increased GLI1 protein and transcript relative to EV controls (Supplementary Fig. 9C). Surprisingly, 67NR KD cells showed no significant change in GLI1 protein or transcript levels compared with EV controls (Supplementary Figs. 9D, 10). The divergent behavior of 4T07 and 67NR cells can be attributed, at least in part, to their intrinsic molecular phenotypes. While 4T07 cells exhibit a basal-like/triple-negative phenotype, characterized by higher proliferative, invasive, and mesenchymal features [39], 67NR cells display a luminal B/ER⁺ profile, associated with epithelial differentiation and limited metastatic potential [40, 41].

**Fig. 6:**
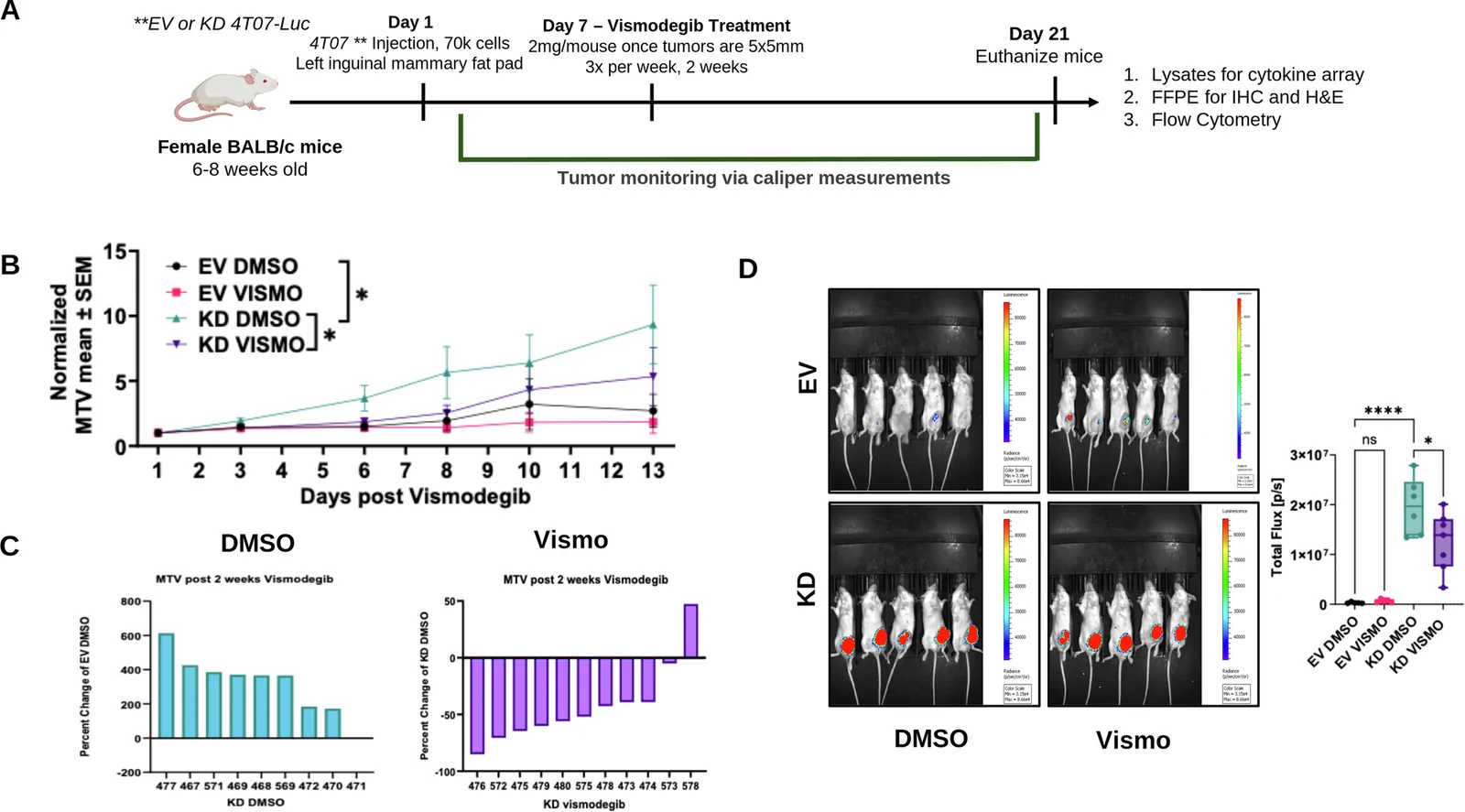
Merlin knockdown promotes primary tumor growth and alters therapeutic response in an orthotopic 4T07 breast cancer model. **A** Schematic of the animal experimental workflow. Luciferase-labeled 4T07 cells (empty vector, EV, or Merlin knockdown, KD) were orthotopically injected into the left inguinal mammary fat pad of female BALB/c mice (6–8 weeks old; 70,000 cells per mouse). Tumor growth was monitored using caliper measurements and bioluminescence imaging (BLI). Vismodegib (2 mg per mouse) was administered orally three times per week for 2 weeks, beginning when tumors reached approximately 5 × 5 mm. Tumor tissues were collected at endpoint for downstream analyses, including cytokine array, immunohistochemistry (IHC), hematoxylin and eosin (H&E) staining, and flow cytometry. **B** Normalized mean tumor volume (MTV) over 14 days after treatment initiation in mice bearing 4T07 tumors (EV or Nf2 KD) treated with vehicle (DMSO) or Vismodegib. For each tumor, MTV was normalized to its day 1 value. **C** Percentage change in tumor volume, calculated relative to the respective DMSO controls. KD tumors demonstrated a more substantial reduction in volume compared to EV tumors after Vismodegib treatment. **D** Representative bioluminescence imaging (BLI) at endpoint shows markedly higher signal in mice bearing Nf2 KD 4T07 tumors versus EV controls. Quantification of ROI bioluminescence from orthotopic tumors (EV or Nf2 KD) treated with DMSO or Vismodegib demonstrates that Nf2 knockdown significantly elevates tumor signal, while Vismodegib reduces signal in Nf2 KD tumors but not in EV tumors. Box and whisker plots display median, interquartile range, and range; each dot represents one mouse. Statistical analysis: Data are presented as mean ± SEM. Unpaired two-tailed t-tests or one-way ANOVA were used where appropriate. *p < 0.05, ****p < 0.0001; ns not significant.

Next, we undertook further analysis to query if Hh activation presents a dependency of Merlin-deficient tumors. 4T07 (EV and KD) cells stably expressing luciferase were implanted orthotopically in the inguinal mammary fat pad (Fig. 6A). Merlin-deficient tumors exhibited a consistent trend of accelerated growth by both caliper measurement and bioluminescence imaging (BLI). Following Vismodegib initiation, normalized MTV (relative to day 1 of Vismodegib treatment) rose steadily in KD tumors and diverged from EV controls between days 6 and 13. Vismodegib had little effect on EV tumors but significantly curtailed KD growth (Fig. 6B; Supplementary Fig. 11A). Waterfall plots depicting percent change in MTV and MTD showed consistent increase in tumor growth of KD DMSO versus EV DMSO, with a broad leftward shift (growth restraint) in KD Vismodegib-treated tumors, consistent with vulnerability to Hh inhibition upon Merlin loss (Fig. 6C; Supplementary Fig. 11B, C). Endpoint BLI mirrored these findings: KD tumors exhibited intense signals that were reduced by Vismodegib, whereas EV signals remained low and unaltered (Fig. 6D).

We conducted similar analysis with EV or *Nf2* KD 67NR cells (Supplementary Fig. 12A). KD tumors were consistently larger than EV counterparts (Supplementary Fig. 12B). Longitudinal measurements indicated that KD tumors expanded more rapidly than EV controls, with clear divergence in MTV from day 18 onward (Supplementary Fig. 12C). In contrast to the robust drug sensitivity observed in 4T07 tumors, Vismodegib produced a modest, non-significant growth delay in *Nf2*-deficient 67NR tumors while remarkably restraining EV tumor outgrowth, suggestive of reduced reliance on canonical Hh despite Merlin loss. In summary, while Merlin loss accelerated tumor growth in both models, the basal-like 4T07 tumors displayed vulnerability to Hh inhibition, suggestive of a subtype-specific therapeutic dependency.

### The immunosuppressive tumor milieu of Merlin-deficient tumors is remodeled by Hedgehog pathway inhibition

**Fig. 7:**
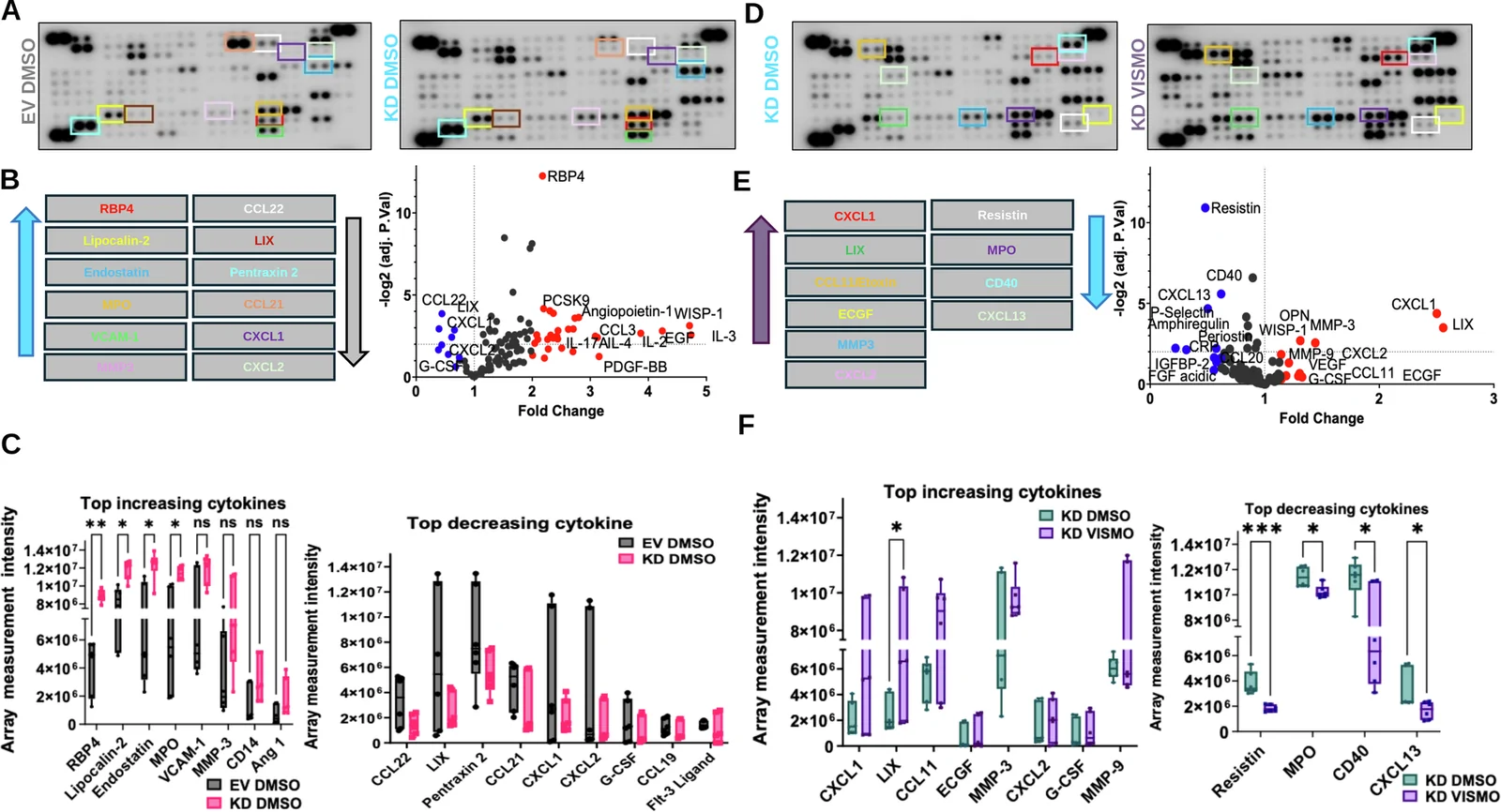
Loss of Merlin alters cytokine signaling in the tumor microenvironment of orthotopic 4T07 breast tumors. **A** Relative cytokine expression was profiled in orthotopic 4T07 tumors with or without Merlin knockdown (KD). Tumor lysates (200 µg per sample; *n* = 3 per group) were analyzed using Mouse XL Cytokine Array membranes. Representative array membranes are shown for EV and KD tumors under DMSO treatment. **B** Volcano plot and cytokine summary of KD DMSO versus EV DMSO tumors. The x-axis depicts log₂ fold change, and the y-axis represents −log10 adjusted *p* value. Cytokines significantly upregulated in KD DMSO tumors (red dots; upward blue arrow). Downregulated cytokines (blue dots; downward gray arrow). **C** Quantitative cytokine profiling revealed that Merlin KD upregulated numerous cytokines involved in myeloid recruitment, matrix remodeling, and tumor-promoting inflammation, including RBP4, Lipocalin-2, Endostatin, MPO, VCAM-1, MMP-3, CD14, and Angiopoietin-1. Conversely, Merlin KD tumors exhibited suppression of cytokines such as CCL22, LIX, Pentraxin 2, Flt3 Ligand, G-CSF, and CXCL family members. *n* = 3 membranes per group. **D** The right panels display cytokine expression profiles in Merlin KD tumors treated with Vismodegib (KD VISMO) relative to DMSO-treated controls (KD DMSO), demonstrating the impact of Hedgehog pathway inhibition on the tumor cytokine milieu. Selected cytokines exhibiting the most pronounced expression changes are outlined with colored boxes. *n* = 3 membranes per group. **E** Volcano plot comparing KD VISMO versus KD DMSO tumors. Cytokines upregulated following Vismodegib treatment are shown as red dots (upward violet arrow), while downregulated cytokines are shown as blue dots (downward light blue arrow). **F** In KD tumors, Vismodegib treatment further modulated cytokine expression. Cytokines, including CXCL1, LIX, CCL11, ECGF, MMP-3, CXCL2, G-CSF and MMP-9 were increased following Hedgehog inhibition, while Resistin, Myeloperoxidase, CD40, and CXCL13 were significantly decreased. All quantitative data are presented as mean ± SEM. Statistical significance was determined using multiple two-tailed Student’s t-tests. Significance thresholds: *p < 0.05, **p < 0.01, ***p < 0.001.

Hh inhibition reshapes the TIME of breast cancer [42, 43]. Building on the sensitivity of 4T07 KD tumors to Hh inhibition (Fig. 6, Supplementary Figs. 11 and 12), we first assessed the cytokine profile of 4T07 EV and KD tumors treated with vehicle control (DMSO) or Vismodegib. In the vehicle-control groups, relative to EV tumors, the KD tumors showed significant upregulation of factors linked to myeloid recruitment and matrix remodeling (RBP4, Lipocalin-2, Endostatin, MPO, VCAM-1, MMP-3, CD14 and Angiopoietin-1) and suppression of cytokines which are associated with immune cell trafficking and stromal inflammatory signaling (CCL22, LIX, Pentraxin 2, FLT3 Ligand, G-CSF and CXCL family members) (Fig. 7A–C). The differential enrichment of cytokines impinged upon TNF, MAPK, and PI3K-AKT signaling, IL-1 and IL-2 family signaling, and HIF-1 signaling (Supplementary Fig. 13A).

Vismodegib treatment elicited changes in the cytokine profile of KD tumors marked by an increase in inflammatory and matrix remodeling mediators (CXCL1, LIX, CCL11, ECGF, MMP-3, CXCL2, G-CSF, and MMP-9) and decreased inflammatory and immunoregulation-associated factors (Resistin, Myeloperoxidase, CD40, and CXCL13) (Fig. 7D–F), with corresponding enrichment of chemokine signaling, IL-17, and IL-1 pathways, and cytokine-cytokine receptor interaction (Supplementary Fig. 13B).

**Fig. 8:**
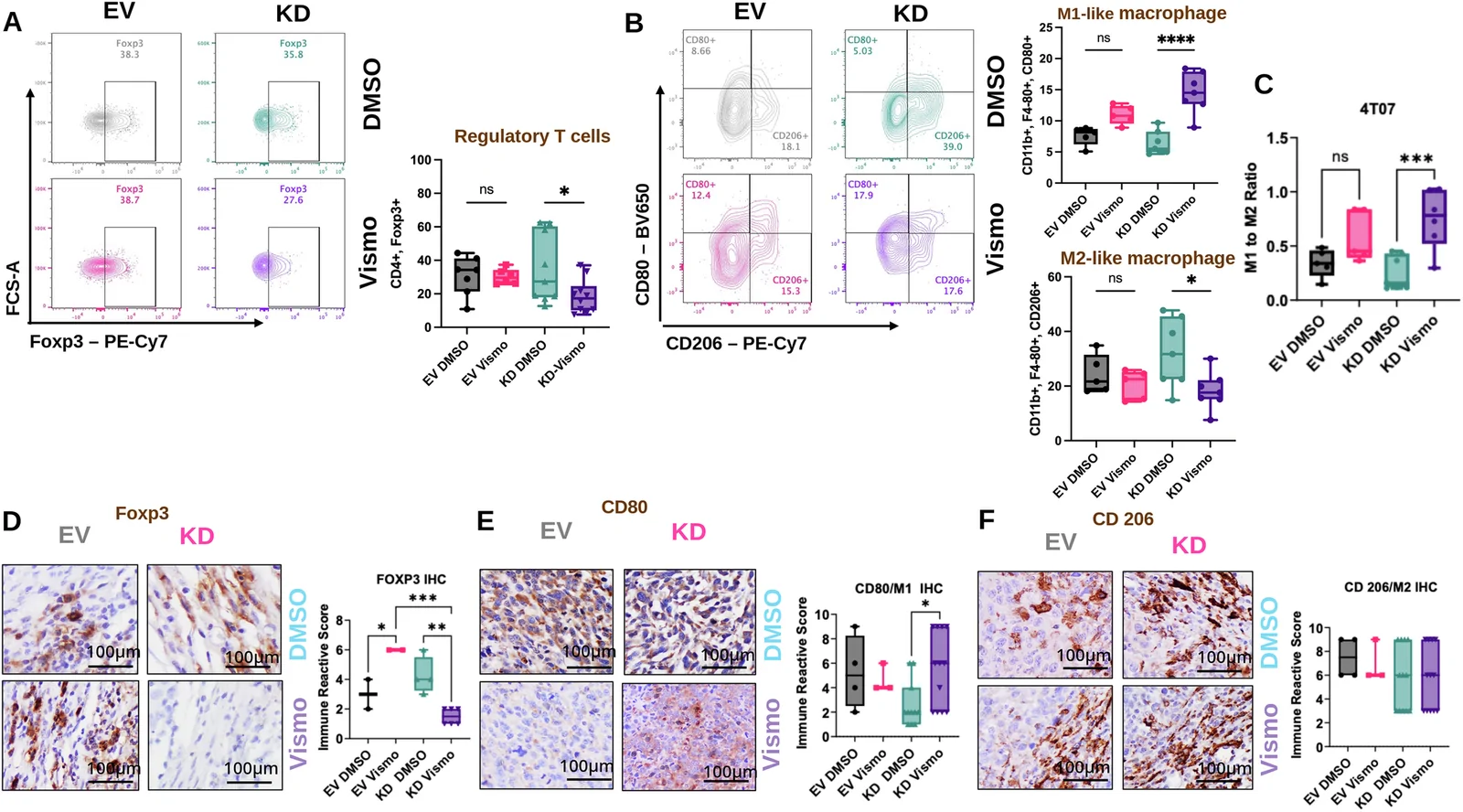
Merlin loss reprograms the immune microenvironment in 4T07 breast tumors and is partially reversed by Hedgehog pathway inhibition. **A** Representative flow cytometry plots showing quantification of Foxp3⁺CD4⁺ regulatory T cells (Tregs). No significant difference was observed between Merlin KD tumors treated with DMSO and EV controls. In contrast, Vismodegib treatment significantly reduced Treg abundance in Merlin KD tumors. **B** Representative double-gating flow cytometry analyses of tumor-infiltrating macrophages from orthotopic 4T07 tumors. Quantification of CD11b⁺F4/80⁺CD80⁺ M1-like macrophages was performed using double gating (Live⁺ → CD45⁺ → CD11b⁺ → F4/80⁺ → CD80⁺ CD206-). Quantification of CD11b⁺F4/80⁺CD206⁺ M2-like macrophages using the same double-gating strategy (Live⁺ → CD45⁺ → CD11b⁺ → F4/80⁺ → CD80- CD206+) showed that these cells were enriched in Merlin KD DMSO-treated tumors compared with EV controls but were significantly reduced after Vismodegib treatment. **C** The M1/M2 macrophage ratio in 4T07 tumors was assessed by flow cytometry under EV and Merlin KD conditions with or without Vismodegib treatment. While both treated tumors showed a trend toward increased M1-like polarization with Vismodegib, statistical significance was observed only in treated KD tumors. **D** Immunohistochemistry demonstrated nuclear Foxp3 expression, visualized as brown chromogenic staining, confirming the presence of Tregs within the tumor microenvironment. Foxp3⁺ Tregs were higher in Merlin KD DMSO tumors compared to EV DMSO and were markedly reduced following Vismodegib treatment, in agreement with flow cytometric quantification. **E** Immunohistochemistry revealed a modest increase of CD80 expression in EV DMSO tumors relative to Merlin KD DMSO, while Merlin KD tumors treated with Vismodegib exhibited a significant increase in CD80⁺ M1-like macrophages, characterized by intensified brown surface staining. This observation was consistent with flow cytometry results, which showed a significant increase in CD11b⁺F4/80⁺CD80⁺ M1-like cells in the same group. **F** CD206⁺ M2-like macrophages, visualized by brown surface staining, showed no change between Merlin KD DMSO and EV DMSO tumors and remained unchanged following Vismodegib treatment, with no statistically significant difference. Statistical analysis: Data are presented as mean ± SEM. Unpaired two-tailed t-tests or one-way ANOVA were used where appropriate. Significance thresholds: *p < 0.05, **p < 0.01, ***p < 0.001, ****p < 0.0001, ns not significant.

We next assessed the tumors for the immunosuppressive cells, namely, regulatory T cells (Tregs) and anti-inflammatory M2-like macrophages. In 4T07 tumors, *Nf2* knockdown did not perturb the relative abundance of CD4⁺ Foxp3⁺ T cells (Tregs) (Fig. 8A), CD206⁺ M2-like macrophages, or inflammatory CD80⁺ M1-like macrophages (Fig. 8B). Vismodegib elicited a remarkable reduction in the abundance of Tregs and M2-like macrophages in the KD tumors (Fig. 8A, B) simultaneous with a significant increase in tumor-infiltrating M1-like macrophages in KD tumors, with little effect in EV tumors (Fig. 8B). Proportionately examined, Vismodegib-treated KD tumors exhibited a significant increase in inflammatory macrophages (Fig. 8C).

Substantiating these assessments, we also evaluated the tumor tissues by IHC. We stained the tissue sections for Foxp3 to visualize Tregs. In alignment with flow cytometry-immunophenotyping, staining the tumor tissues for Foxp3 demonstrated higher abundance of Tregs in KD tumors and their depletion following Vismodegib therapy (Fig. 8D). Abundant CD206⁺ macrophages were observed in both groups with a treatment-dependent increase in CD80⁺ cells only in KD tumors (Fig. 8E, F). Taken together, these results demonstrate that Vismodegib reprograms the TIME of 4T07 *Nf2*-deficient tumors by decreasing Tregs and suppressive macrophages and boosting inflammatory macrophages. In the 67NR model, Merlin status did not impact Tregs or macrophages (Supplementary Fig. 14A, B). Vismodegib significantly reduced Tregs and M2-like macrophages in both EV and KD tumors and significantly increased CD80⁺ M1-like macrophages (Supplementary Fig. 14A, B). Hh inhibition markedly altered the influx of M1 macrophages to EV and KD tumors with a concomitant reduction in M2 macrophages. Vismodegib significantly increased the proportion of M1 macrophages relative to M2 macrophages only in the KD tumors (Supplementary Fig. 14C). We also assessed the impact of Vismodegib on tumor-infiltrating Th1 cells and cytotoxic T cells, both of which orchestrate anti-tumor responses. Interestingly, Vismodegib treatment elicited an increase in CD4+ Tbet+ Th1 cells in 4T07 KD and 67NR KD tumors (Supplementary Figs. 15A and 16A) without remarkable changes in CD8+ Granzyme B+ cytotoxic T cells (Supplementary Figs. 15B and 16B). Collectively, the data indicate that Merlin loss is associated with features of an immunosuppressive TIME potentially linked to Hh activity characterized by Treg expansion, an M2-like macrophage bias, and cytokine milieu that promotes myeloid recruitment and matrix remodeling.

## Discussion

Merlin, the protein product of *NF2*, is recognized as a pivotal tumor suppressor in multiple tissue contexts [44]. Large-scale pan-cancer analyses demonstrate frequent Merlin loss across epithelial malignancies, including breast carcinoma, malignant mesothelioma, papillary renal cell carcinoma, and pancreatic ductal adenocarcinoma [45]. Here, we integrated clinical-omics data with functional and experimental studies in two immunocompetent breast cancer models, triple-negative 4T07 and luminal B/ER⁺ 67NR cells, and showed that Merlin deficiency is associated with oncogenic signaling activation, reshaping the TIME, and subtype-specific therapeutic vulnerability.

Merlin loss consistently promoted aggressive tumor behaviors, as reflected by increased migration, invasion, 3D spheroid expansion, and colony formation. Analogous increases in proliferative and migratory capacity have also been documented when Merlin is lost in hepatocellular carcinoma [46], glioblastoma [47], and meningioma [48].

A consistent pattern emerging from the bioinformatic interrogation of patient cohorts and our in vitro and in vivo experiments is the linkage between Merlin loss and an EMT program. Within TCGA and CPTAC datasets, tumors harboring low Merlin expression displayed significant enrichment of Hallmark EMT, MYC, and E2F gene sets. Merlin deficiency led to downregulation of epithelial determinants (e.g., CDH1) and upregulation of mesenchymal markers (VIM, CDH2) together with EMT-driving transcription factors (SNAI2, ZEB2) [49]. These alterations were functionally reflected as accelerated wound closure and increased invasion, hallmarks of a migratory invasive state consistent with EMT [50].

In vivo, in murine models of breast cancer, Merlin deficiency accelerated tumor growth, characterized by increased angiogenesis and resistance to apoptosis. This behavior mirrors patterns reported in other malignancies, including mesothelioma, glioblastoma, hepatocellular carcinoma, and non-small cell lung cancer, where Merlin deficiency similarly fuels unchecked growth, vascular remodeling, and survival advantages [44, 51]. As such, given the aggressive biology associated with Merlin loss and the scarcity of effective targeted options, especially in breast cancer, there is a pressing need to explore new therapeutic strategies.

The present study demonstrates that the EMT-associated transcriptional and functional changes driven by Merlin loss provide a biological framework for understanding its impact on tumor progression in vivo. Merlin-deficient tumors harbored increased immunosuppressive tumor-infiltrating Foxp3⁺ Tregs and CD206⁺ M2-like macrophages, a pattern reminiscent of other Merlin-deficient malignancies [52]. Complementing the immune cell shifts that we observed, Merlin KD upregulated myeloid-recruiting, inflammatory, and matrix remodeling mediators (e.g., RBP4, Lipocalin-2, Endostatin, MPO, VCAM-1, MMP-3, CD14, and Angiopoietin-1) while decreasing mediators for inflammatory T cell trafficking and stromal inflammatory signaling (e.g., CCL22, LIX, Pentraxin 2, FLt3 Ligand, G-CSF, and CXCL family members), consequently generating an immunosuppressive milieu with a myeloid skewed state. Functionally, this signature is associated with enhanced myeloid recruitment and M2 polarization (via factors such as M-CSF and IL-10) while ANGPT1/VCAM-1 supports endothelial activation and myeloid tethering. Similar cytokine landscapes have been reported in pleural mesothelioma [53] and vestibular schwannoma [52], where Merlin deficiency is usually associated with macrophage-rich, lymphocyte-poor, and dysfunctional T-cell microenvironments [52]. Aligning with our previous study [7], bulk RNA-seq and GSEA uncovered enrichment of Hh-related gene sets in Merlin-deficient triple-negative 4T07 tumors, which associated with sensitivity to Vismodegib, supporting the potential therapeutic relevance of this clinically approved SMO inhibitor for Merlin-deficient triple-negative breast cancer. Supporting this possibility, Vismodegib alleviated the immunosuppressive tumor milieu by depleting Tregs and M2-like (pro-tumor) macrophages while promoting M1-like (anti-tumor) macrophages, thereby fostering a more inflammatory and immune-stimulating microenvironment. These current findings extend prior foundational work by demonstrating that pharmacologic Hh blockade induces immune reprogramming across mammary tumors representing basal-like and luminal B/ERα⁺ subtypes [43, 54].

In both models, Hh inhibition led to reduction of tumor-infiltrating Treg cells and M2-like macrophages, expansion of M1-like macrophages, and a cytokine profile aligned with an inflammatory tumor immune environment, demonstrating that Hh pathway blockade can alleviate the immunosuppression induced by Merlin loss.

## Supplementary information

Supplementary Information

## Statement

During the preparation of this work, the authors used GPT-4 to improve the readability of the manuscript. After using this tool/service, the authors reviewed and edited the content as needed and take full responsibility for the content of the publication.
