# Supplementary material for: Merlin deficiency supports an immunosuppressive milieu in breast cancer
Source: Cell Death Discov. 2026 Jul 9;12:397. doi: 10.1038/s41420-026-03223-7 (PMC13633259; doi:10.1038/s41420-026-03223-7)
Supplement: Supplementary file 1 [file 41420_2026_3223_MOESM1_ESM.docx]

**SUPPLEMENTARY FIGURE LEGENDS**

**Supp Figure 1. Pan Cancer and Breast Cancer Analyses Link Merlin Deficiency to Poor Prognosis and Aggressive Disease.**

(A) Pan cancer analysis of NF2 genetic alterations across The Cancer Genome Atlas (TCGA) datasets. The bar graph displays the frequency of NF2 alterations, including mutations, deep deletions, and amplifications across various tumor types. The highest frequencies of NF2 alterations were observed in mesothelioma (MESO), uterine carcinosarcoma (UCS), and glioblastoma multiforme (GBM), suggesting broad relevance of NF2 dysregulation in oncogenesis.
(B) Reverse-phase protein array (RPPA) based Merlin expression levels across multiple solid tumors. Protein levels of Merlin were compared across tumor types. Notably, Merlin protein was significantly downregulated in breast (BRCA), prostate (PRAD), and endometrial (UCEC) cancers relative to normal tissue controls, indicating translational suppression in several epithelial malignancies.
(C–E) Kaplan–Meier survival analyses across pan cancer cohorts stratified by Merlin expression. Patients with low Merlin expression (red) exhibited significantly reduced progression-free interval (PFI; C) and overall survival (OS; D, E) in pan cancer and pan cancer analysis of whole genomes compared to those with high Merlin expression (blue), highlighting the prognostic significance of Merlin loss in a pan cancer context. Log-rank p-values and hazard ratios (HR) are indicated on each plot.

**Supp Figure 2. Merlin loss predicts poor clinical outcomes and drives EMT, oncogenic, and stemness related transcriptional reprogramming in breast cancer.** (A) Post-Progression Survival (PPS): Even after disease progression, low NF2 mRNA expression (red) remained associated with shorter survival duration (HR = 0.69; 95% CI: 0.55–0.88; P = 0.0023).
(B) Regional recurrence-free survival analysis based on NF2 copy number status from the Caldas and Chin CGH datasets. Poor survival was observed in patients with low NF2 copy number (red), reinforcing a potential link between genomic loss of NF2 and locoregional disease recurrence.
(C) Gene Set Enrichment Analysis (GSEA, Hallmark database) of low NF2 expression tumors revealed enrichment of oncogenic and stemness-associated pathways, including MYC targets, epithelial–mesenchymal transition (EMT), E2F targets, glycolysis, and TNFα signaling via NF-κB. Bubble size indicates gene set size; color scale reflects nominal P-values.
(D) Reactome GSEA analysis of transcriptomic data from breast tumors with low NF2 expression confirmed metabolic and epigenetic reprogramming in Merlin deficient tumors, highlighting enrichment of glycosylation precursor biosynthesis, miRNA biogenesis, RNA silencing, cholesterol metabolism, and cell motility programs.
(E) KEGG GSEA analysis of transcriptomic data from breast tumors with low NF2 expression identified enrichment of metabolic and signaling programs.

(F) Gene set enrichment analysis (GSEA) of bulk RNA-seq and proteomic datasets from TCGA breast invasive carcinoma (via Xena Browser) and heatmap (genes listed are strongly associated with EMT among the ranked genes) demonstrated significant enrichment of EMT associated transcriptional programs in Merlin deficient tumors (P = 0.0122; normalized enrichment score [NES] = 1.424).

**Supp Figure 3: Merlin knockdown reprograms the transcriptome and induces an EMT program in 4T07 and 67NR cells, as demonstrated by bulk RNA-seq.**

(A, B) Heatmaps showing unsupervised hierarchical clustering of differentially expressed genes (DEGs) in 4T07 (A) and 67NR (B) cells. Nf2 loss induced widespread transcriptional reprogramming characterized by upregulation of genes associated with oncogenic, metabolic, and inflammatory signaling (yellow), and downregulation of differentiation and adhesion related transcripts (purple), indicating a profound shift in cellular state.
(C) Differential expression analysis of bulk RNA-seq comparing Nf2 knockdown (KD) and empty vector (EV) control cells for EMT markers.

All quantitative data are presented as mean ± SEM. Statistical significance was determined using a two-tailed Student’s t-test for RNA-seq data.
Significance thresholds: *p < 0.05, **p < 0.01, ***p < 0.001, ****p < 0.0001.

**Supp Figure 4. Merlin loss drives oncogenic transcriptional reprogramming across breast cancer cell lines.**

(A, B) Volcano plots depicting DEGs of bulk RNA-seq data in Nf2 KD versus control cells in 4T07 (A) and 67NR (B) models.

(C, D) Gene ontology (GO) analysis of DEGs in Nf2-KD 4T07 and 67NR cells. Complementary Hallmark GSEA refined these signals: in 4T07 (C), MYC and E2F target programs and TGF-β and KRAS signaling were significantly enriched; in 67NR (D), enrichment included E2F and MYC programs together with mTORC1 signaling and TNF-α/NF-κB.

**Supp Figure 5. Reactome and KEGG Analyses Reveal Pathway Enrichment Following Merlin Silencing in 4T07 and 67NR Breast Cancer Cells.**

(A–D) Gene Set Enrichment Analysis (GSEA) of bulk RNA-seq data was conducted using Reactome and KEGG gene sets to further characterize the transcriptomic consequences of Nf2 knockdown (KD) in 4T07 and 67NR murine breast cancer cells compared to empty vector (EV) controls.
(A, B) Reactome pathway enrichment analysis in 4T07 (A) and 67NR (B) Nf2 knockdown (KD) cells. In 4T07 cells, Reactome analysis revealed enrichment of pathways related to cilium assembly, cholesterol biosynthesis, and G-protein–coupled receptor (GPCR) signaling. In contrast, 67NR KD cells exhibited enrichment of DNA damage repair, interleukin signaling, and complement activation pathways, indicating a predominance of immune-related and proliferative signaling reprogramming.

(C, D) KEGG pathway enrichment analysis in 4T07 (C) and 67NR (D) Nf2 knockdown cells. In 4T07 cells, enriched pathways included steroid and glycosphingolipid biosynthesis as well as cell cycle related programs. In contrast, 67NR cells showed enrichment of DNA replication, cell cycle, and extracellular matrix (ECM) receptor interaction pathways.

**Supp Figure 6. Independent shRNA-mediated Merlin knockdown confirms epithelial–mesenchymal transition (EMT) induction in 4T07 and 67NR breast cancer cells.**

(A) Representative immunoblot analysis confirming Merlin knockdown using an independent shRNA in 4T07 and 67NR cells, with corresponding changes in E-cadherin and Vimentin expression. β-actin was used as a loading control.

(B) qRT-PCR analysis of EMT-related genes following Merlin knockdown (KD) versus empty vector (EV). Data are presented as mean ± SEM. Statistical significance was determined using a two-tailed Student’s t-test. Significance thresholds: *p < 0.05, ***p < 0.001, ****p < 0.0001.

**Supp Figure 7. Merlin loss enhances migratory, invasive, and proliferative capacities in 4T07 and 67NR breast cancer cells using independent shRNA constructs.**

(A, B) Representative images and quantification of migrated cells from migration assays in 4T07 (A) and 67NR (B) cells following Merlin knockdown (KD) utilizing a non-overlapping shRNA, showing increased migration compared to empty vector (EV) controls.

(C, D) Representative images and quantification of invaded cells from Transwell Matrigel invasion assays in 4T07 (C) and 67NR (D) cells following Merlin knockdown (KD) demonstrating increased invasive capacity compared to EV controls.

(E) Representative images of EV and Merlin KD 4T07 3D spheroids following transfection with a second shRNA. Quantification of spheroid circularity demonstrates decreased circularity and increased protrusive in KD cells.

(F) Representative images of EV and Merlin KD 4T07 3D colony formation assay following transfection with a second shRNA. Quantification of colonies demonstrate increased number of colonies in KD cells.

Data are presented as mean ± SEM. Statistical analysis was performed using unpaired two-tailed Student’s t-test. *p < 0.05, **p < 0.01 and ****p < 0.0001

**Supp Figure 8. Merlin loss in orthotopic 67NR tumors drives tumor growth and promotes angiogenesis, proliferation, and apoptosis resistance.**

(A) Tumor growth kinetics of mice injected with control (EV, black) or Nf2 knockdown (KD, pink) 67NR cells. 1 × 10⁶ EV or KD 67NR cells were orthotopically implanted into the right mammary fat pad of female BALB/c mice. Mean tumor volume (MTV) was measured at the indicated points post-injection.
(B) Immunohistochemical staining of Merlin in 67NR derived tumors revealed a substantial reduction in chromogenic intensity in Merlin knockdown (KD) tumors compared to empty vector (EV) controls. This pronounced loss of Merlin expression in KD tumors was quantitatively confirmed by Immune Reactive Score (IRS) analysis (***p < 0.001), validating effective NF2 suppression.
(C) CD31 staining demonstrated an enrichment of vascular structures in KD tumors, with an increase in both staining intensity and vessel number. Quantitative assessment showed a significant rise in CD31 expression in the absence of Merlin (p < 0.01), reflecting enhanced neovascularization.
(D) Analysis of PCNA, a nuclear marker of DNA synthesis and proliferation, exhibited heightened nuclear staining in KD tumors. The observed increase in proliferative index was statistically supported by IRS scoring (p < 0.01), consistent with accelerated tumor cell cycling driven by Nf2 depletion.
(E) Detection of apoptotic cells via Annexin V immunostaining revealed diminished staining in KD tumors relative to controls, suggesting that Merlin loss may attenuate apoptotic signaling. IRS scoring supported a downward trend in Annexin V positivity, indicative of impaired apoptosis.
(F) AgNOR staining (silver-stained nucleolar organizer regions) revealed no difference in nucleolar number between EV and KD 67NR tumors.
Data are presented as mean ± SEM. Statistical comparisons were performed using appropriate tests; *p < 0.05; **p < 0.01.

**Supp Figure 9. Merlin loss activates Hedgehog signaling in a lineage dependent manner.**
(A–B) Gene Set Enrichment Analysis (GSEA) of bulk RNA-seq data comparing Nf2 knockdown (KD) versus empty vector (EV) cells revealed subtype-dependent Hedgehog pathway activation. Hedgehog signaling gene sets curated in the Molecular Signatures Database (MSigDB) were significantly enriched in 4T07 Nf2 KD cells (A) and 67NR Nf2 KD cells (B).
(C) Immunoblotting (α-tubulin loading control) and RT-qPCR analysis demonstrated increased GLI1 expression in 4T07 Nf2 KD cells compared with EV controls. Bar graph shows RT-qPCR quantification of Gli1 transcript, normalized to EV.
(D) Immunoblotting (tubulin loading control) and RT-qPCR analysis of GLI1 in 67NR Nf2 KD cells revealed no significant change compared with EV controls. Bar graph shows RT-qPCR quantification of Gli1 transcript, relative to EV.
Data represent mean ± SEM from three independent experiments. Statistical significance was assessed using a two-tailed Student’s t-test, with p < 0.05 considered significant.

**Supp Figure 10. Independent targeting validates lineage-dependent GLI1 regulation following Merlin loss.**

(A) Immunoblot and RT-qPCR analysis of GLI1 expression in 4T07 cells following NF2 knockdown (KD). β-actin was used as a loading control.

(B) Immunoblot and RT-qPCR analysis of GLI1 expression in 67NR cells following NF2 knockdown (KD). β-actin was used as a loading control.

Data are presented as mean ± SEM. Statistical analysis was performed using unpaired two-tailed Student’s t-test. *p < 0.05.

**Supp Figure 11. Enhanced Tumor Growth Inhibition by Vismodegib in Merlin Deficient Orthotopic 4T07 Breast Cancer Models.**

(A) Representative images of excised tumors from each treatment group. Tumor size was assessed using a standardized ruler for scale.
(B) Percentage change in mean tumor diameter (MTD), calculated relative to respective EV DMSO controls. KD tumors demonstrated a greater increase in tumor width compared to EV tumors.
(C) Percentage change in mean tumor diameter (MTD), calculated relative to respective KD DMSO controls. KD tumors treated with Vismodegib showed a greater reduction in tumor mass compared to EV tumors.

**Supp Figure 12. Merlin Knockdown Promotes Tumor Growth in the 67NR Orthotopic Breast Cancer Model.**

(A) Experimental design: 67NR breast cancer cells with either control empty vector (EV) or Merlin knockdown (KD) were orthotopically injected into the right inguinal mammary fat pad of female BALB/c mice. When tumors reached approximately 5 × 5 mm, mice were treated orally with either DMSO (vehicle) or Vismodegib (2 mg per mouse) three times per week for ~3 weeks. Tumors were harvested at the study endpoint for flow cytometry and downstream analyses.
(B) Representative tumor images of excised tumors from each group. A standardized ruler is shown for scale.
(C) Tumor growth kinetics: Tumor progression was assessed by mean tumor volume (MTV) over time. Merlin KD tumors exhibited a faster growth rate compared to EV controls. Vismodegib treatment reduced tumor size metrics in the EV group but had no significant effect in KD tumors.

**Supp Figure 13. Merlin Loss and Hedgehog Pathway Inhibition Reshape Cytokine Signaling and Inflammatory Programs in 4T07 Breast Tumors.**

A) Reactome, KEGG and WikiPathways enrichment analyses of differentially expressed cytokines in Merlin KD tumors (KD DMSO) versus empty vector controls (EV DMSO).

(B) Reactome, KEGG and WikiPathways enrichment analyses of differentially expressed cytokines in KD Vismodegib versus KD DMSO tumors.

**Supp Figure 14. Merlin loss remodels the immune landscape within the orthotopic 67NR breast cancer model.**
Tumor-infiltrating immune cells were analyzed by sequential gating of live single cell suspensions (Live⁺ → CD45⁺ → CD11b⁺ → F4/80⁺), followed by identification of macrophage subsets expressing CD80⁺ (M1-like) or CD206⁺ (M2-like). Regulatory T cells (Tregs) were identified by gating Live⁺ → CD45⁺→ CD4⁺ → Foxp3⁺ within the lymphocyte compartment.
(A) Representative flow cytometry plots of Foxp3⁺CD4⁺ Tregs. Treg abundance was comparable between Merlin KD DMSO tumors and EV controls but was significantly reduced in KD tumors following Vismodegib treatment in both groups.
(B) Representative double gating flow cytometry analyses of tumor-infiltrating macrophages from orthotopic 67NR tumors. A significant increase in M1-like macrophages abundance was observed following Vismodegib treatment in both EV and Merlin KD tumors, consistent with enhanced pro-inflammatory polarization. Quantification of CD11b⁺F4/80⁺CD206⁺ M2-like macrophages using the same gating approach showed that M2 macrophage levels were comparable between Merlin KD DMSO treated tumors and EV controls. Vismodegib treatment reduced M2 macrophages abundance in both groups, with a more pronounced reduction in Merlin KD tumors.
(C) The M1/M2 macrophage ratio in 67NR tumors was quantified under EV and Merlin KD conditions with or without Vismodegib treatment. While both treated tumors showed a trend toward increased M1 polarization with Vismodegib, statistical significance was observed only by in treated KD tumors.
Statistical analysis: Data are presented as mean ± SEM. Statistical significance was determined using one-way ANOVA; Significance thresholds: *p < 0.05, **p < 0.01, ***p < 0.001, ****p < 0.0001.

**Supp Figure 15. CD4⁺ and CD8⁺ T Cell Changes Following Merlin Loss in 4T07 Tumors.**

(A) Representative plots and quantification of CD4⁺T-bet⁺ (Th1) T cells in EV and KD tumors ± Vismodegib.

(B) Representative plots and quantification of CD8⁺GzmB⁺ cytotoxic T cells in EV and KD tumors ± Vismodegib.

Statistical analysis: Data are presented as mean ± SEM. one-way ANOVA were used where appropriate. Significance thresholds: **p < 0.01.

**Supp Figure 16. T Cell Changes Following Merlin Loss in 67NR Tumors.**

(A) Representative plots and frequency of CD4⁺T-bet⁺ T cells in EV and KD tumors ± Vismodegib.

(B) Representative plots and frequency of CD8⁺GzmB⁺ T cells in EV and KD tumors ± Vismodegib.

Data are presented as mean ± SEM.
